# Postoperative Radiotherapy for pT1- and pT2-Classified Squamous Cell Carcinoma of the External Auditory Canal †

**DOI:** 10.3390/cancers16234026

**Published:** 2024-11-30

**Authors:** Cindy H. Nabuurs, Wietske Kievit, Charles (René) Reinier Leemans, Conrad F. G. M. Smit, Michiel W. M. van den Brekel, Robert J. Pauw, Bernard F. A. M. van der Laan, Jeroen C. Jansen, Martin Lacko, Weibel W. Braunius, Chunfu Dai, Xunbei Shi, Giovanni Danesi, Jan Bouček, Daniele Borsetto, Shravan Gowrishankar, Romain Kania, Clément Jourdaine, Thijs T. G. Jansen, Jolanda Derks, Tim Dijkema, Robert P. Takes, Henricus (Dirk) P. M. Kunst

**Affiliations:** 1 Department of Otorhinolaryngology and Head and Neck Surgery—Academic Alliance Skull Base Pathology Radboudumc & MUMC+, Radboud University Medical Center, 6525 GA Nijmegen, The Netherlands; wietske.kievit@radboudumc.nl (W.K.); thijs.jansen@radboudumc.nl (T.T.G.J.); jolanda.derks@radboudumc.nl (J.D.); robert.takes@radboudumc.nl (R.P.T.); dirk.kunst@radboudumc.nl (H.P.M.K.); 2 Rare Cancers, Radboud Institute for Health Sciences, 6525 EZ Nijmegen, The Netherlands; 3 Department of Health Evidence, Radboud University Medical Center, 6525 GA Nijmegen, The Netherlands; 4 Department of Otolaryngology—Head and Neck Surgery, Amsterdam University Medical Centers, VU University, 1081 HV Amsterdam, The Netherlands; cr.leemans@amsterdamumc.nl (C.R.L.); cf.smit@amsterdamumc.nl (C.F.G.M.S.); 5 Department of Head and Neck Surgery and Oncology, Netherlands Cancer Institute—Antoni van Leeuwenhoek, 1066 CX Amsterdam, The Netherlands; m.vd.brekel@nki.nl; 6 Department of Otorhinolaryngology and Head and Neck Surgery, Erasmus Medical Center, 3015 GD Rotterdam, The Netherlands; r.pauw@erasmusmc.nl; 7 Department of Otorhinolaryngology and Head and Neck Surgery, University Medical Center of Groningen, University of Groningen, 9713 GZ Groningen, The Netherlands; b.van.der.laan@haaglandenmc.nl; 8 Department of Otorhinolaryngology Head and Neck Surgery, Haaglanden Medical Center, 2512 HH The Hage, The Netherlands; 9 Department of Otorhinolaryngology and Head and Neck Surgery, Leiden University Medical Center, 2333 ZA Leiden, The Netherlands; j.c.jansen@lumc.nl; 10 Department of Otorhinolaryngology and Head and Neck Surgery—Academic Alliance Skull Base Pathology Radboudumc & MUMC+, Maastricht University Medical Center, 6229 HX Maastricht, The Netherlands; martin.lacko@mumc.nl; 11 Department of Head and Neck Surgical Oncology, University Medical Center, Utrecht Cancer Center, 3584 CG Utrecht, The Netherlands; w.braunius@umcutrecht.nl; 12 Department of Otology & Skull Base Surgery, Eye Ear Nose & Throat Hospital, Fudan University, Shanghai 200437, China; cfdai66@163.com (C.D.); xunbei1991@163.com (X.S.); 13 Department of Otorhinolaryngology and Skull Base Microsurgery-Neurosciences, Azienda Socio Sanitaria Territoriale Papa Giovanni XXIII, 24127 Bergamo, Italy; gdanesi@asst-pg23.it; 14 Department of Otorhinolaryngology, Head and Neck Surgery, The First Faculty of Medicine, Charles University in Prague, University Hospital Motol, 150 06 Prague, Czech Republic; jboucek99@seznam.cz; 15 Department of Otorhinolaryngology, Head and Neck Surgery, Cambridge University Hospitals NHS Foundation Trust, Cambridge CB2 0QQ, UK; daniele.borsetto@addenbrookes.nhs.uk (D.B.); shavran.growishankar@nhs.net (S.G.); 16 Department of Head and Neck Surgery, Lariboisière University Hospital, 75010 Paris, France; romain.kania@aphp.fr (R.K.); clement.jourdaine@aphp.fr (C.J.); 17 Department of Radiation Oncology, Radboud University Medical Center, 6525 GA Nijmegen, The Netherlands; tim.dijkema@radboudumc.nl

**Keywords:** temporal bone, squamous cell carcinoma, disease-free survival, radiotherapy, treatment

## Abstract

Background: There is no consensus regarding the indication for postoperative radiotherapy (PORT) for T1- and T2-classified squamous cell carcinoma (SCC) of the external auditory canal (EAC) even with negative surgical margins. This study aimed to evaluate whether PORT provides additional benefits for these cases. Methods: We collected retrospective data from fourteen international hospitals, including resected pT1- and pT2-classified EAC SCC with negative surgical margins. Results: A total of 112 early-stage radically resected EAC SCC were included, with 48 patients receiving PORT. The 5-year DFS of T1- and T2-classified EAC SCC treated with PORT was not statistically significantly different (92.9% and 76.9%, respectively) compared to the group treated without PORT (100% and 90.9%, respectively; *p*-values of 0.999 and 0.526, respectively). EAC SCC treated with PORT more frequently exhibited perineural and angioinvasive growth. Eighteen patients experienced side effects related to radiotherapy, of which one patient developed osteoradionecrosis. Conclusions: Our study suggests that PORT for early-stage radically resected EAC SCC should only be considered in selected cases with perineural, infiltrative growth or angioinvasive growth, and with a close margin. This approach helps mitigate the negative impact on quality of life and the risk of side effects associated with radiotherapy.

## Simple Summary

Squamous cell carcinoma (SCC) of the external auditory canal (EAC) is a rare malignancy, and its disease-free survival (DFS) is strongly associated with surgical margins and tumor classification. Early-stage EAC SCC has higher survival rates than advanced-stage EAC SCC, suggesting that less aggressive treatment strategy might be sufficient. However, the additional benefits of post-operative radiotherapy (PORT) for early-stage EAC SCC that has been completely resected are unclear. Our retrospective study aimed to evaluate whether PORT offers additional benefits for such cases. Our results showed no significant difference in 5-year DFS between patients treated with PORT and those without PORT. EAC SCC treated with PORT more frequently exhibited perineural and angioinvasive growth compared to those without PORT. Thirty-eight percent of the patients undergoing PORT experienced side effects of radiotherapy. These findings suggest that PORT should be reserved for selected cases with high-risk features to minimize side effects and preserve quality of life.

## 1. Introduction

Squamous cell carcinoma (SCC) of the external auditory canal (EAC) is an exceptionally rare malignancy. The modified Pittsburgh tumor classification is strongly associated with the disease-free survival (DFS). Patients with T3- or T4-classified EAC SCC exhibit a 5-year DFS ranging from 35% to 84.4% [1,2,3,4,5,6], while those with T1- or T2-classified EAC SCC demonstrate a higher 5-year DFS ranging from 65% to 100% [1,2,3,4,5]. Consequently, a less aggressive treatment strategy might suffice for T1- and T2-classified EAC SCC, referred to as early-stage EAC SCC. If post-operative radiotherapy (PORT) does not significantly enhance the oncologic outcomes for early-stage EAC SCC, its use might expose patients unnecessarily to potential complications, such as osteoradionecrosis, secondary tumors, scar breakdown and infections. Conversely, inadequate treatment can be life-threatening, because salvage therapy of recurrent or residual disease is associated with a high morbidity and mortality [7].

Surgical removal of the tumor remains the first choice of treatment for EAC SCC. However, the use of PORT in early-stage EAC SCC is a matter of debate. Most surgeons recommend PORT only for T2–T4 EAC SCC with negative tumor features (e.g., close or positive margins, multiple regional node metastases, nodal extracapsular extension) [2,5,8,9,10,11,12,13,14]. In a meta-analysis of Oya et al. [15], 45% of stage I and 68% of stage II EAC SCC received PORT, indicating that many early-stage EAC SCC also undergo PORT. This meta-analysis included 21 observational studies with 170 patients with early-stage EAC SCC. However, it lacked correction for confounding by indication, such as surgical margins. Negative surgical margins are known to be strongly associated with higher survival outcomes compared to positive surgical margins [1,16,17,18]. Nevertheless, the existing literature does not conclusively establish whether PORT significantly benefits patients with early-stage EAC SCC with negative surgical margins in terms of survival outcome. Moreover, it remains unclear which additional factors may influence the survival outcome of radically removed early-stage EAC SCC.

Our retrospective study aims to address whether PORT provides additional benefits for radically removed pT1- and pT2-classified EAC SCC by observing the impact of PORT and exploring other potential prognostic factors affecting the DFS outcomes of early-stage EAC SCC and side effects of PORT.

## 2. Methods

### 2.1. Database

Approval was obtained from the medical ethics committee of Radboud University Medical Center (number 2017-3397, dated 1 November 2017), and participating centers adhered to their local medical ethics committee requirements.

Initially, a nationwide Dutch cohort study was conducted, including patients who were treated with curative intent for their primary EAC SCC at one of the eight Dutch head and neck oncological centers between 1975 and 2017. Two nationwide systems were used to identify the patients: ICD-code, “International Statistical Classification of Diseases and Related Health Problems” and PALGA, “Pathologisch-Anatomisch Landelijk Geautomatiseerd Archief”: a nationwide pathology registry. The diagnosis was subsequently verified manually by examining the medical records.

This nationwide database was combined with international retrospective data from patients treated for EAC SCC at the Eye and ENT Hospital of Fudan University (Shanghai, China) between 2005 and 2018, Papa Giovanni XXIII hospital (Bergamo, Italy) between 2012 and 2019, Ospedale Università di Padova (Padova, Italy) between 2014 and 2017, Motol University Hospital (Prague, Czech Republic) between 2011 and 2020, Cambridge University Hospitals NHS Foundation Trust (Cambridge, United Kingdom) between 2006 and 2018, and Lariboisière Hospital (Paris, France) between 2004 and 2021.

### 2.2. Patient Selection

This study included only patients with pathological (*p*) T1- or T2-classified EAC SCC, according to the modified Pittsburgh classification system [19], and negative surgical margins. Negative surgical margin was defined as the pathologist not finding any cancer cells at the edge of the tissue. Patients were excluded if the primary tumor location was not the EAC (e.g., external ear canal, temporal bone or middle ear); if the histologic subtype was not SCC; if the EAC SCC was not the primary tumor; if the medical records were too limited for tumor staging; if patients were not treated surgically; if it was unknown whether the patient received PORT; or if they did not receive surgical treatment with curative intent. Patients were also excluded if they were treated by local resection or piecemeal resection combined with local application of 5-fluorouracil to enhance data homogeneity.

### 2.3. Radiotherapy Protocol

The radiotherapy protocol for EAC SCC was available from six of the fourteen participating centers. Among these, one center used proton therapy, two centers used Intensity-modulated radiation therapy (IMRT), one used Volumetric modulated arc therapy (VMAT), and one transitioned from Three-dimensional conformal radiation therapy (3D CRT) (1990–2003) to IMRT (until about 2007) and finally to VMAT. Target areas varied, with five centers focusing exclusively on the primary tumor site, while one center targeted the primary tumor site, ipsilateral parotid region, and ipsilateral neck levels II-V. The total radiation dose ranged from 56 to 74 Gy for the local region and 0 and 70 Gy for regional region, depending on lymph node metastasis or whether a neck dissection was performed. Fraction sizes varied between 2 and 2.2 Gy per session.

### 2.4. Analyses

Descriptive data analysis was performed for pT1- and pT2-classified EAC SCC subgroups with and without PORT. Per subgroup, we studied the following factors: age, gender, lymph node metastases, type of surgical techniques, neck dissection, parotidectomy events, adverse histological features consisting of perineural growth, angioinvasion and infiltrative growth, and surgical margin. Possible associations between categorical variables were evaluated using the chi-squared test, and continuous variables were compared using either the independent samples *t*-test or the Wilcoxon rank-sum test, based on the data’s distribution. An event was defined as local and/or regional, as residual disease, recurrence of the disease, or death. The DFS was defined as the number of months between the date of the surgery until the date an event was diagnosed or the last date of follow-up without any event. Unfortunately, correction for confounders by indication was not feasible, due to small subgroups. The DFS outcomes were analyzed for pT1- and pT2-classified EAC SCC treated with and without PORT using the Kaplan–Meier survival analysis. Differences in 5-year DFS outcomes between the subgroups treated with and without PORT were assessed using Cox regression analyses. All data analyses were conducted in R version 3.4.3 [20]. In all analyses, a probability (*p*) value < 0.05 was considered statistically significant.

## 3. Results

This study included a total of 56 patients with pT1-classified and 56 patients with pT2-classified EAC SCC, all with negative surgical margins. Table 1 provides an overview of the baseline characteristics for each subgroup. The median age for all patients was 65.5 years (range: 15–92 years). Three patients with pT1-classified tumors and five patients with pT2-classifed tumors had pathologically proven lymph node metastasis (N+). Locations of the lymph node metastasis were unknown. Forty-eight patients received PORT, with the radiation volume being only local in seven patients and locoregional in three patients; for thirty-eight patients, data were missing. Eleven patients had residual disease or recurrence of the EAC SCC; two patients with a pT1-classified EAC SCC were treated with PORT, seven patients with a pT2-classified EAC SCC were treated with PORT and two patients with a pT2-classified EAC SCC were treated without PORT. The location of recurrence or residual disease could not be correlated with radiation volume due to missing data. During the follow-up period (median 45 months; range 0–180 months), seven patients died from the EAC SCC and 15 patients due to other causes. The 5-year DFS outcome of pT1N0- and pT2N0-classified EAC SCC treated with PORT was comparable to the subgroup that did not receive PORT, with rates of 95.5% and 82.6%, respectively, and a *p*-value of 0.101.

### 3.1. Treatment Strategies

Table 1 and Table 2 show the applied treatments per subgroup. Lateral temporal bone resection (LTBR) was the most common treatment for both pT1- and pT2-classified EAC SCC with or without PORT. Nineteen pT1-classified EAC SCC were surgically removed using local resection, such as sleeve resection. pT1N0- and pT2N0-classified EAC SCC with PORT more often underwent elective neck dissection (43.8%, 7/16 patients and 29.2%, 7/29 patients, respectively) compared to subgroups without PORT (10.8%, 4/37 pT1-classified EAC SCC and 22.2%, 6/27 pT2-classified EAC SCC). pT1-classified EAC SCC were less frequently treated with additional parotidectomy (41%, 23/56 patients) compared to pT2-classified EAC SCC (76%, 46/60 patients). Conversely, additional parotidectomy was more common in the pT1-classified EAC SCC subgroup with PORT (58%, 11/19 patients) compared to without PORT (32%, 12/37 patients). Patients with a pT1-classified EAC SCC treated in one of the centers received more frequently a neck dissection (8/9 patients) and a parotidectomy (8/9 patients) compared to the other centers (neck dissection: 5/38 patients and parotidectomy: 11/38 patients). Conversely, an additional parotidectomy was less common in the pT2-classified EAC SCC subgroup with PORT (69%, 20/29 patients) compared without PORT (85%, 23/27 patients). Patients with a pT2-classified EAC SCC treated in four of the centers received more frequently a neck dissection (8/13 patients) and a parotidectomy (10/13 patients) compared to the other ten centers (neck dissection: 1/9 patients and parotidectomy: 1/8 patients and one unknown). All patients with clinically proven lymph node metastasis underwent a parotidectomy and/or a neck dissection. The margin was 0.1–1 mm in one patient, 1–5 mm in 21 patients, ≥ 5 mm in 16 patients and unknown in 78 patients. PORT was not given to the patient with a surgical margin of 0.1–1 mm; it was given in 13 patients with a surgical margin of 1–5 mm and in 9 patients with a surgical margin of ≥ 5 mm.

Table 2 shows that 16 patients with pT1N0-classified EAC SCC received PORT. The radiation volume for these patients was local in four, locoregional in two and unknown in ten. Twenty-four patients with a pT2N0-classified EAC SCC received PORT, with the radiation volume being local in three patients, locoregional in one patient and unknown in 22 patients. PORT was given to all patients with pathologically proven lymph node metastasis, but data on the exact radiation volume in this subgroup are missing.

No patient was treated with additional chemotherapy.

### 3.2. Histological Features

Unfortunately, not all patient data were available to explore whether specific histological features were related to the choice of receiving PORT. Table 1 shows that of the eight patients with perineural growth, only one did not receive PORT. Only the subgroup pT1-classified EAC SCC with PORT had angioinvasion, 21.1% (4/19). Of the nine patients with infiltrative growth, only two did not receive PORT. Pearson’s chi-squared test showed no statistically significant differences in the number of these various adverse histological features with and without PORT for pT1- and pT2-classified EAC SCC.

### 3.3. Five-Year DFS Outcomes

**Figure 1:**
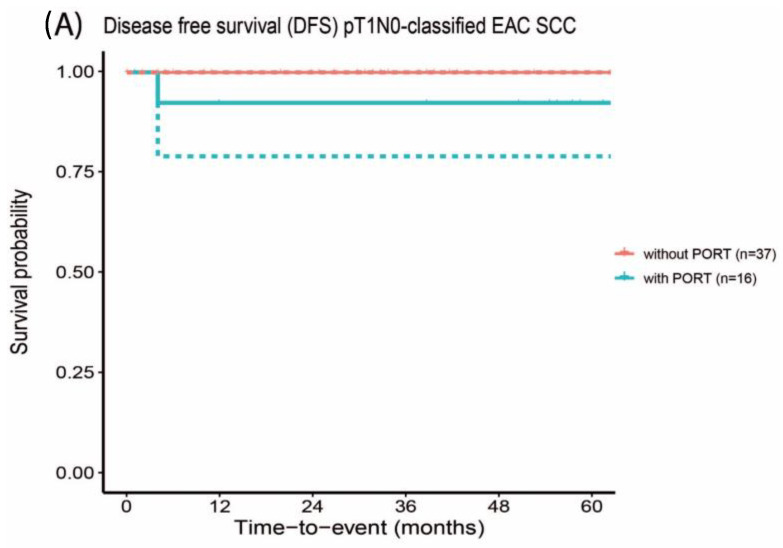

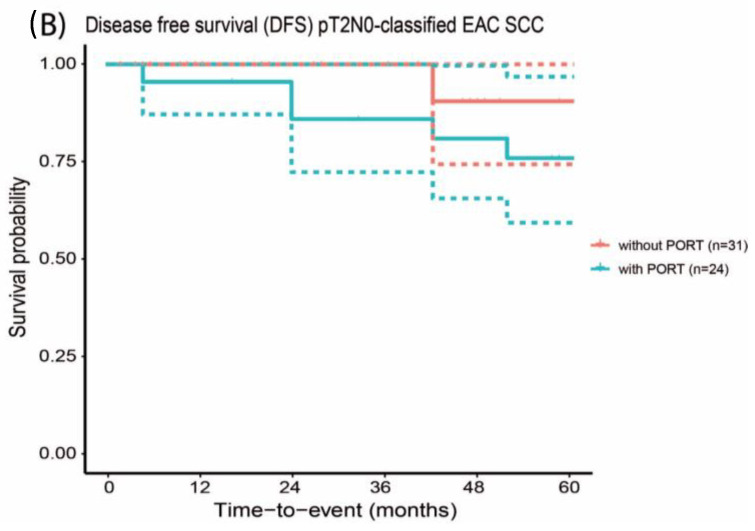
Disease-free survival outcome of early-stage EAC SCC with negative surgical margins with and without postoperative radiotherapy. The confidence interval is depicted by the dotted lines.

The 5-year DFS outcome of pT1N0-classified EAC SCC with PORT was comparable to pT1N0-classified EAC SCC that did not receive PORT (92.9% and 100%, respectively, with a *p*-value of 0.999), Figure 1A. Similarly, the 5-year DFS outcome of pT2N0-classified EAC SCC that received PORT was comparable with and not statistically significantly different from the subgroup that did not receive PORT (76.9% and 90.9%, respectively, *p*-value of 0.526), Figure 1B.

All EAC SCC with lymph node metastasis received PORT. The 5-year DFS outcome of pT1N+-classified EAC SCC was 50% and 92.9% for the pT1N0-classified EAC SCC. This difference was not statistically significant (hazard ratio (HR) 6.481, confidence interval (CI) 0.405–103.8, *p*-value of 0.187). For the pT2N+-classified EAC SCC, the 5-year DFS was 80%, and for pT2N0-classified EAC SCC it was 76.9%. This difference was also not statistically significant (HR 0.831, CI 0.100–6.918, *p*-value 0.864).

The patient with a 0.1–1 mm surgical margin had a follow-up of 43 months without recurrence. The 5-year survival was 94.7% (CI 85.2–100) for 1–5 mm and 84.4 (CI 66.6–100) % for ≥ 5 mm. The differences were not statistically significant, with a *p*-value of 0.998.

When examining the 5-year DFS outcomes of various histological features, the 5-year DFS outcomes of pT1-classified EAC SCC treated with PORT with perineural growth (66.7%, CI 30–100%; HR 6.928, CI 0.390–123.1, *p*-value 0.187) and/or angioinvasion (50%, CI 12.5–100%; HR 8.832, CI 0.543–145.9, *p*-value 0.128) seem to be poorer compared to without perineural growth (91.7%, CI 77–100%) and/or without angioinvasion (91.7%, CI 77–100%). Within the subgroup pT1-classified EAC SCC treated with PORT, similar 5-year DFS outcomes were seen for the tumors with and without infiltrative growth (100%). However, within the subgroup pT2-classified EAC SCC treated with PORT, the 5-year DFS outcomes of tumors with infiltrative growth (75%, CI 43–100%; HR 1.457, CI 0.132–16.07, *p*-value 0.759) seem to be poorer compared to tumors without infiltrative growth (90.9%, CI 75–100%). In our available data, none of the pT2-classified EAC SCC had angioinvasion, and none of the pT2-classified EAC SCC treated without PORT had perineural growth.

### 3.4. Side Effects and Complications

Among the patients who received PORT, 47% (18/38) experienced side effects due to the radiotherapy. The most common reported side effect was erythematous skin lesions, also known as radiation dermatitis (*n* = 7), which is transient. Three patients developed dysphagia, two patients developed xerostomia, one patient had mucositis, and in one patient, treatment was complicated by osteoradionecrosis.

## 4. Discussion

The main goal of this study was to contribute to the ongoing discussion on the potential benefits of PORT for early-stage EAC SCC. The impact of PORT on radically resected pT1- and pT2-classified EAC SCC in terms of the 5-year DFS outcomes and side effects of PORT were studied in an international cohort of patients. Based on our retrospective study, the 5-year DFS outcome was slightly, though statistically not significantly, poorer for patients with pT1N0- and pT2N0-classified EAC SCC that received PORT compared to those without PORT. This trend was also observed in various surgical margins within the subgroup of T1-classified tumors. Histologic features for applying PORT were often perineural and infiltrative growth and angioinvasion. The survival outcomes of EAC SCC with these histological features were poorer (83.3% and 88.9% for perineural growth and infiltrative growth, respectively) compared EAC SCC without these adverse histological features, despite additional PORT (94.4% and 97.7% for perineural growth and infiltrative growth, respectively, not statistically significantly different). The 5-year DFS outcomes were still poorer (50% for pT1N+-classified and 80% for pT2N+-classified EAC SCC) compared to EAC SCC without lymph node metastasis treated with PORT (92.9% for pT1N0-classified and 88.8% for pT2N0-classified EAC SCC).

Although some studies showed outcomes comparable to our results [15,19,21], there were other studies showing that PORT was associated with higher survival outcomes [8,15]. However, none of these studies considered positive surgical margins, surgical strategies, lymph node metastasis or adverse histological features. The DFS outcomes in head and neck cancer are strongly correlated with surgical margins [22,23]. The Royal College of Pathologists defines head and neck tumors with surgical margins of < 1 mm as ‘involved’, 1–5 mm as ‘close’ and > 5 mm as ‘clear’ [24]. Unfortunately, our data on the surgical margin were largely missing. From the available data, no clear relation was observed between the surgical margin and the benefit of PORT. Furthermore, the 5-year DFS outcome was slightly, though not statistically significantly, poorer for patients with pT1-classified EAC SCC that received PORT compared to those without PORT. PORT may have contributed to a smaller difference in DFS outcomes between these subgroups. Therefore, PORT is recommended for EAC SCC with involved surgical margins (< 1 mm) and could be considered for EAC SCC with close surgical margins (1–5 mm), especially in the presence of adverse histological features such as perineural, infiltrative or angioinvasive tumor growth.

The added value of PORT is also influenced by the surgical techniques. The type of surgical resection can affect the risk of positive surgical margins, which is again correlated with lower survival outcomes [5,7,13,19,21,25,26,27]. Controversy exists regarding the preferred surgical techniques for early-stage EAC SCC; local resection (e.g., local canal resection and sleeve resection) versus lateral temporal bone resection (LTBR). Some surgeons prefer local resection to preserve hearing [12,28]. However, the risk of positive surgical margins is higher with local resection, leading to increased recurrence risk and reduced survival outcomes despite PORT [28]. This is supported by Zhang et al., who show that 53.8% of T1- and T2-classified tumors treated with local resection had positive margins and 46.2% of them had recurrence. Their study also shows that T1- and T2-classified tumors treated with LTBR resulted in 0% positive margins and 0% recurrence. Therefore, most surgeons prefer LTBR for T1- and T2-classified tumors [10,13,29,30,31,32,33,34]. With a LTBR, the cortical bone of the EAC is totally resected, resulting in radical resection in the majority of patients. Despite clear margins and PORT, our results indicate that the 5-year DFS outcomes of LTBR may be higher than local resection, prompting consideration of other factors influencing survival outcomes for early-stage tumors.

One of the factors that influences the survival outcomes of patients with EAC SCC negatively is lymph node metastasis [35], making it an indication for PORT. However, the risk of occult nodal metastases is relatively low, especially for pT1-classified EAC SCC (0%) and pT2-classified EAC SCC (7%) [36]. Our data show no improvement in the 5-year DFS of patients without nodal metastases treated with additional PORT (92.9% and 76.9% for pT1N0- and pT2N0-classified EAC SCC, respectively) compared to patients without additional PORT (100% and 90.9% for pT1N0- and pT2N0-classified EAC SCC, respectively).

Histological features may also impact survival outcomes [37,38,39,40,41]. This study shows that the DFS outcome of pT1-classified EAC SCC with perineural growth and/or angioinvasion is poorer compared to pT1-classified EAC SCC without these histological features, despite being treated with PORT. The DFS outcome of pT2-classified EAC SCC with infiltrative growth also seems to be poorer compared to pT2-classified EAC SCC without infiltrative growth, despite PORT. The 5-year DFS outcomes of pT1-classified EAC SCC with infiltrative growth were 100% for both subgroups treated with and without PORT. In our data, none of the pT2-classified EAC SCC had angioinvasive growth, and none of the pT2-classified EAC SCC treated without PORT had perineural growth. Although no statistically significant differences were found in the number of these various adverse histological features between subgroups with and without PORT for pT1- and pT2-classified EAC SCC, this is probably due to the small subgroups. Several studies, particularly on oral cavity SCC, have demonstrated a negative correlation between perineural growth and survival outcomes [37,38,39,40,41]. Tumors with perineural growth may have poorer margin control due to tumor spread along nerves beyond surgical margins at the time of treatment [39]. Some studies also show that vascular invasion is significantly correlated with poorer survival outcomes [40,41]. Vascular invasion may leave tumor cells in the patient after surgery. To our knowledge, our study is the first study that explored also the impact of infiltrative growth on survival outcome and shows that these three histological features (perineural, angioinvasive and infiltrative growth) may result in poorer survival outcomes for early-stage EAC SCC. For tumors with these features, PORT might be of added value to improve a potentially poor survival outcome. However, the exact effect of PORT remains unproven.

Another factor to consider in the evaluation of PORT is side effects (calculated and often transient) or complications due to radiotherapy. Among our study population who received PORT, 18 out of 35 patients experienced side effects due to radiotherapy, consisting of dermatitis, dysphagia, xerostomia and mucositis. One patient developed osteoradionecrosis. For thirteen patients treated with PORT, data about side effects and complications were missing.

A study by Niska et al. [42] showed that their entire study population experienced at least one grade 2 adverse event during radiotherapy of the head and neck region (n = 65). Their study reported higher incidence rates of various side effects: 98% had dermatitis, 56–83% dysgeusia, 60–98% odynophagia and 48–80% xerostomia. Most of these side effects will be transient, however. The impact of xerostomia also depends on the volume of salivary gland tissue in or near the radiation volume. Side effects due to PORT of EAC SCC studied by Nagaro et al. [43] were dermatitis, osteoradionecrosis, temporomandibular joint complaints and stenosis of the EAC. Long-term side effects of radiotherapy for head and neck cancers negatively impact overall quality of life [42]. If PORT has limited value for patients in terms of survival outcomes, the risk of long-term sequalae of PORT and their negative impact on quality of life may outweigh the benefits. If PORT is necessary, the risk of complications and side effects and the negative impact on quality of life can be reduced with conformal radiation techniques such as intensity-modulated radiotherapy [44].

### Limitations of the Study

Our study has also some limitations that need acknowledgement. Studies on EAC SCC, including our current study, are often retrospective cohorts, because of the rarity of this disease. Retrospective studies come with the inherent risk of confounding that is challenging to fully address. This challenge is particularly pronounced when dealing with relatively small study populations. Despite our study having a relatively high number of participants for early-stage EAC SCC, the sample size remains insufficient for in-depth subgroup analysis or correction for confounding by indication; for example, the influence of the surgical margin which seems to be a possible confounder based on the match analyses in Table 1. However, data on the surgical margin were largely missing, and the known surgical margin varied between 0.3 mm and > 5 mm. The limited number of patients makes it challenging to draw robust conclusions. Nevertheless, by exploring the histological features, we tried to explore their impact on the choice of whether PORT was given or not and its impact on the survival outcomes. Unfortunately, not all data on histological features were available, resulting in a potential risk of bias. Despite these limitations, our study stands as one of the largest cohorts of EAC SCC patients, providing valuable data for clinical decision making.

## 5. Conclusions

Based on our exploratory retrospective study, the 5-year DFS outcome was slightly, though statistically not significantly, poorer for patients with pT1N0- and pT2N0-classified EAC SCC that received PORT compared to those without PORT. The DFS outcome of the subgroup treated with PORT is not statistically significantly different from the subgroup without PORT, which may be due to the PORT, as this subgroup more frequently had adverse histological features.

Therefore, PORT for all cases of early-stage EAC SCC does not seem feasible. This is reinforced by the negative impact of radiotherapy on quality of life and the high risk of side effects associated with it. However, PORT for early-stage EAC SCC could be considered if the tumor has perineural, angioinvasive or infiltrative growth, especially in conjunction with close surgical margins (1–5 mm), and it should be given if lymph node metastasis is present or if the surgical margins are < 1 mm.

## Figures and Tables

**Table 1:** Descriptive analyses per subgroup.

		pT1					pT2				
		PORT		No PORT			PORT		No PORT		
		N = 19	5-Year DFS (CI)	N = 37	5-Year DFS (CI)	*p*-Value	N = 29	5-Year DFS (CI)	N = 27	5-Year DFS (CI)	*p*-Value
Age (years)	Median (min–max)	69.0 (45–83)		65.0 (15–86)		0.282	67.0 (46–92)		64 (35–86)		0.143
Gender	Male	11 (57.9%)	87.5 (67–100)	26 (70.3%)	100	0.53	15 (51.7%)	85.1 (68–100)	16 (59.3%)	100 (0–100)	0.767
	Female	8 (42.1%)	85.7 (63–100)	11 (29.7%)	100		14 (48.3%)	70.1 (49–100)	11 (40.7%)	83.3 (58–100)	
pN-classification	N0	16 (84.2%)	92.9 (80–100)	37 (100%)	100	0.063	24 (82.8%)	76.9 (61–97)	27 (100%)	90.9 (75–100)	0.073
	N+	3 (15.8%)	50.0 (12.5–100)	0			5 (17.2%)	80 (52–100)	0		
Surgical technique	Local resection	4 (21.1%)	50.0 (12.5–100)	15 (40.5%)	100	0.517	1 (3.4%)	100	1 (3.7%)	100 (0–100)	0.693
	LTBR	13 (68.4%)	90.9 (75–100)	19 (51.4%)	100		25 (86.2%)	73.7 (58–94)	22 (81.5%)	87.5 (67–100)	
	STBR	1 (5.3%)	100	1 (2.7%)	100		2 (6.8%)	100	1 (3.7%)	100 (0–100)	
	TTBR	1 (5.3%)	100	2 (5.4%)	100		1 (3.4%)	100	3 (11.1%)	100 (0–100)	
Neck dissection	Yes	10 (52.6%)	68.6 (40–100)	4 (10.8%)	100	0.002	9 (31.0%)	77.8 (55–100)	6 (22.2%)	75 (43–100)	0.787
	No	9 (47.4%)	100	32 (86.5%)	100		20 (69%)	77.3 (60–100)	19 (70.4%)	100	
	NA	0		1 (2.7%)	100		0		2 (7.4%)	100	
Parotidectomy	Yes	11 (57.9%)	78.7 (56–100)	12 (32.4%)	100	0.142	20 (69%)	73 55–96)	23 (85.2%)	88.9 (71–100)	0.119
	No	8 (42.1%)	100	24 (64.9%)	100		8 (27.6%)	87.5 (67–100)	2 (7.4%)	100	
	NA	0		1 (2.7%)	100		1 (3.4%)	100	2 (7.4%)	100	
Surgical margin	0.1–1 mm	0		1 (2.7%)	NA	0.028	0		0		NA
	1–5 mm	7 (36.8%)	80 (51.6–100)	7 (18.9%)	100		6	100	0		
	≥5 mm	5 (26.3%)	66.7 (30–100)	7 (18.9%)	100		4	75 (42.6–100)	0		
	NA	7 (36.8%)	100	22 (59.5%)	100		19	72.2 (54.2–96.2)	27	90.9 (75–100)	
Adverse histological features	Perineural growth	5 (26.3%)	66.7 (30–100)	1 (2.7%)	100	0.054	2 (6.9%)	100	0		1
	No perineural growth	13 (68.4%)	91.7 (77–100)	27 (73%)	100		16 (55.2%)	87.5 (73–100)	4 (14.8%)	100	
	Angioinvasion	4 (21.1%)	50.0 (12.5–100)	0		0.038	0		0		NA
	No angioinvasion	14 (73.7%)	91.7 (77–100)	28 (75.7%)	100		18 (62.1%)	88.9 (76–100)	4 (14.8%)	100	
	Infiltrative growth	3 (15.8%)	100	2 (5.4%)	100	0.367	4 (13.8%)	75 (43–100)	0		0.637
	No infiltrative growth	9 (47.4%)	100	23 (62.2%)	100		11 (37.9%)	90.9 (75–100)	4 (14.8%)	100	
Recurrence/Residual		2 † (10.5%)		0		0.194	7 ‡ (24.1%)		0 (0%)		0.162

Abbreviations: CI, confidence interval; DFS, disease-free survival; N0, no lymph node metastasis; N+, proven lymph node metastasis; NA, not available; LTBR, lateral temporal bone resection; STBR, subtotal temporal bone resection; TTBR, total temporal bone resection; PORT, postoperative radiotherapy. †: 1 patient with local recurrence with distant metastasis and 1 patient with distant metastasis. ‡: 2 patients with local recurrences, 2 patients with regional recurrence, 1 patient with intracranial metastasis and 2 missing data of the location of the recurrences.

**Table 2:** Treatment strategy per subgroup.

		pT1N0	pT1N+	pT2N0	pT2N+
		N = 53	N = 3	N = 55	N = 5
Surgical technique	Local resection	17	2	2	0
	LTBR	31	1	46	5
	STBR	2	0	3	0
	TTBR	3	0	4	0
Neck dissection		11	3	14	2
Parotidectomy		20	3	41	5
PORT		16	3	25	5
	Local	4	0	3	0
	Regional	0	0	0	0
	Locoregional	2	0	1	1
	Missing data	10	3	21	4
	Median total dosage (Gy, min–max)	60 (50–66)	NA	60 (50–66)	60 (50–60)
Recurrence/Residual		0	2 †	6	1
	Local	0	1	2	0
	Regional	0	0	1	1
	Distant	0	2	1	0
	Missing	0	0	2	0

Abbreviations: N0, no lymph node metastasis; N+, proven lymph node metastasis; NA, not available; LTBR, lateral temporal bone resection; STBR, subtotal temporal bone resection; TTBR, total temporal bone resection; PORT, postoperative radiotherapy. †: 1 patient with local recurrence with distant metastasis and 1 patient with distant metastasis.

## Data Availability

The data presented in this study are available on request from the corresponding author due to the privacy of patients and medical centers.
